# Relationship Between Arm Swing Angles and Intervertebral Spinal Rotation Angles During Treadmill Walking

**DOI:** 10.7759/cureus.81210

**Published:** 2025-03-25

**Authors:** Tsubasa Yamaguchi, Eizaburo Suzuki, Hiroshi Katoh

**Affiliations:** 1 Department of Physical Therapy, Graduate School, Yamagata Prefectural University of Health Sciences, Yamagata, JPN

**Keywords:** arm swing angle, asymmetry, motion analysis, treadmill walking, vertebral rotation angle

## Abstract

Introduction

Arm swing during walking plays an important role in reducing energy expenditure and enhancing motor control. Arm swing has been traditionally considered to occur due to passive factors associated with spinal rotational movements. However, the relationship between arm swing angles and rotational angles at each spinal level remains unclear. Therefore, this study aimed to investigate the relationship between arm swing angles and spinal rotation angles at each level.

Methods

A total of 21 young, healthy males were included in this study. Measurements were performed during steady-state walking on a treadmill (Split R, SENSTYLE Ltd., Kumamoto, Japan) at a controlled speed. The spine was divided into four levels using a three-dimensional motion analysis system (VICON MX-T, Vicon Motion Systems Ltd., Oxford, UK): upper thoracic (T1-T7), lower thoracic (T7-L1), upper lumbar (L1-L3), and lower lumbar (L3-S). The system calculated the horizontal plane rotation angles at these levels during one gait cycle and the sagittal plane rotation angles of the lateral epicondyles of the left and right upper arms as arm swing angles. Statistical analysis was performed to compare differences in arm swing angles between the left and right arms and in rotation angles between the spinal levels. Additionally, correlation analysis was performed to evaluate the relationship between arm swing angles and spinal rotation angles at each level.

Results

The left arm swing angle was significantly greater than the right arm swing angle. The rotation angle at the T1-T7 level was the lowest among all levels. Additionally, the analysis of the relationship between arm swing angles and spinal rotation angles revealed that the left arm swing angle was significantly positively correlated with the L3-S angle.

Conclusions

Although the lower lumbar vertebrae influence left arm swing during steady-state walking, arm swing is not entirely dependent on the rotational movement of the entire spine and may be influenced by multiple other factors.

## Introduction

Walking is an important activity of daily living. Arm swing in gait is a kinematic feature that plays a crucial role in minimizing energy expenditure, reducing impact forces from the ground, and canceling angular momentum by moving in the opposite direction to the swinging lower limbs [1-3].

Asymmetry in arm swing angles has been reported in patients with stroke and Parkinson’s disease [4,5]. Recently, it has gained attention as an indicator for assessing early signs of neurological disorders. However, asymmetry in arm swing angles has also been reported in healthy individuals [6-8]. Passive factors associated with spinal rotational movements have been reported to be one of the mechanisms of arm swing development [9-11]. In other words, spinal rotational movements accompanying lower limb propulsion generate mechanical energy that is transmitted from the shoulder girdle to the upper limbs, passively inducing arm swing. However, previous studies have defined the rotational angle of specific vertebrae as representative of the movement of the entire spine [9-11], making it difficult to determine the effects of rotational movements between individual segments, such as the thoracic and lumbar vertebrae. Therefore, the relationship between arm swing and spinal rotation angles at each level remains unclear. In addition, walking speed, step length, and walking environment have been reported to affect arm swing angles and spinal rotation angles during walking [12]. In particular, walking speed is an important factor that greatly affects the kinematics of walking, and it is important to examine these dynamics while eliminating the effects of walking speed [13].

Therefore, this study aimed to investigate the relationship between arm swing angles and spinal rotation angles at each level during steady-state walking on a treadmill at a controlled speed. The findings of this study will provide new insights into the mechanism underlying the generation of asymmetric arm swing angles during walking and will serve as valuable basic data for evaluating abnormal gait in patients with neurological disorders, developing rehabilitation treatments to improve walking function, and enhancing activities of daily living (ADL) and quality of life (QOL).

## Materials and methods

Participants

**Figure 1:**
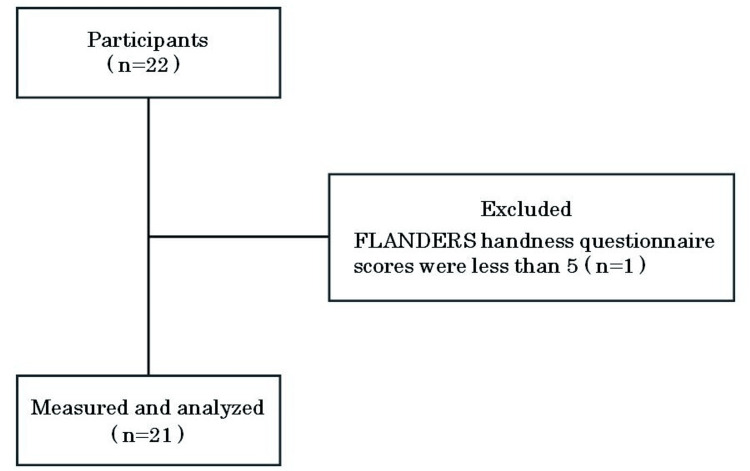
Diagram illustrating the study’s inclusion criteria

A total of 21 young, healthy men were included in this study. The mean age of the participants was 20.2 ± 1.1 years, the mean height was 170.0 ± 7.0 cm, the mean body weight was 63.7 ± 8.5 kg, and the mean body mass index was 22.0 ± 2.5 kg/m². The criteria for inclusion required participants to have no history of psychiatric, neurological, or orthopedic disorders and to be able to walk independently without the use of assistive devices. Additionally, participants needed to demonstrate a preference for right-handedness by scoring five or more on the FLANDERS handedness questionnaire [14], as revealed in Figure 1.

The FLANDERS handedness questionnaire is made up of 10 questions concerning the use of the upper limbs, with responses provided on a three-choice scale: The options "left hand," "both hands," or "right hand" are used for responses. Where the "right hand" receives a score of +1, "left hand" is scored as −1, and "both hands" is scored as 0, the total score determines handedness. A score near +10 signifies strong right-handedness, a score close to −10 signifies strong left-handedness and a score near 0 indicates ambidexterity. The exclusion criteria were subjects with a history of surgery within the past year and those with any pain during walking. This study was approved by the Ethics Committee of Yamagata Prefectural University of Health Sciences (No. 2308-17) and conducted over one year from September 1, 2023, in accordance with the Declaration of Helsinki. Informed consent was obtained from all participants after providing a comprehensive written explanation of the study.

Measurement procedures

**Figure 2:**
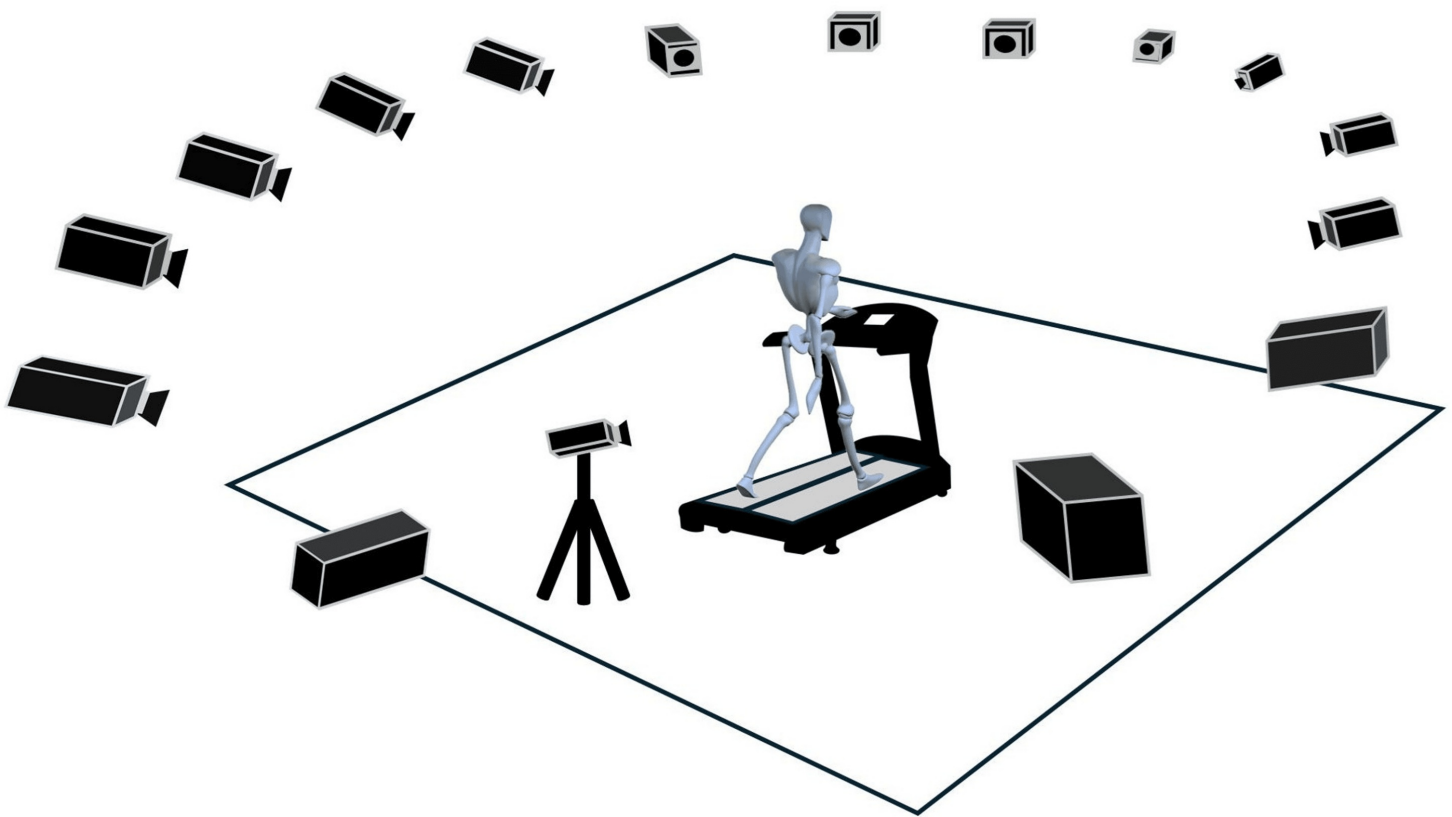
Measurement environment The treadmill was at the center of the measurement room. Fifteen cameras were mounted on the ceiling, whereas one camera was placed on the floor to ensure precise data collection. The fifteen cameras were positioned 3 meters above the floor in a 360° arrangement, and the single camera was placed at a height of approximately 1.3 meters. This image features one element sourced from silhouetteAC [15], which is free of copyright, along with another element designed using 3D animation software (Poser12, Bondware Inc., TN, USA). Image Credits: Tsubasa Yamaguchi.

The measurement task involved steady-state walking on a treadmill at a controlled speed using a split-belt treadmill (Split R, SENSTYLE Ltd., Kumamoto, Japan). Data acquisition was performed using a three-dimensional motion analysis system (VICON MX-T, Vicon Motion Systems Ltd, Oxford, UK) with 16 cameras (Figure 2).

**Figure 3:**
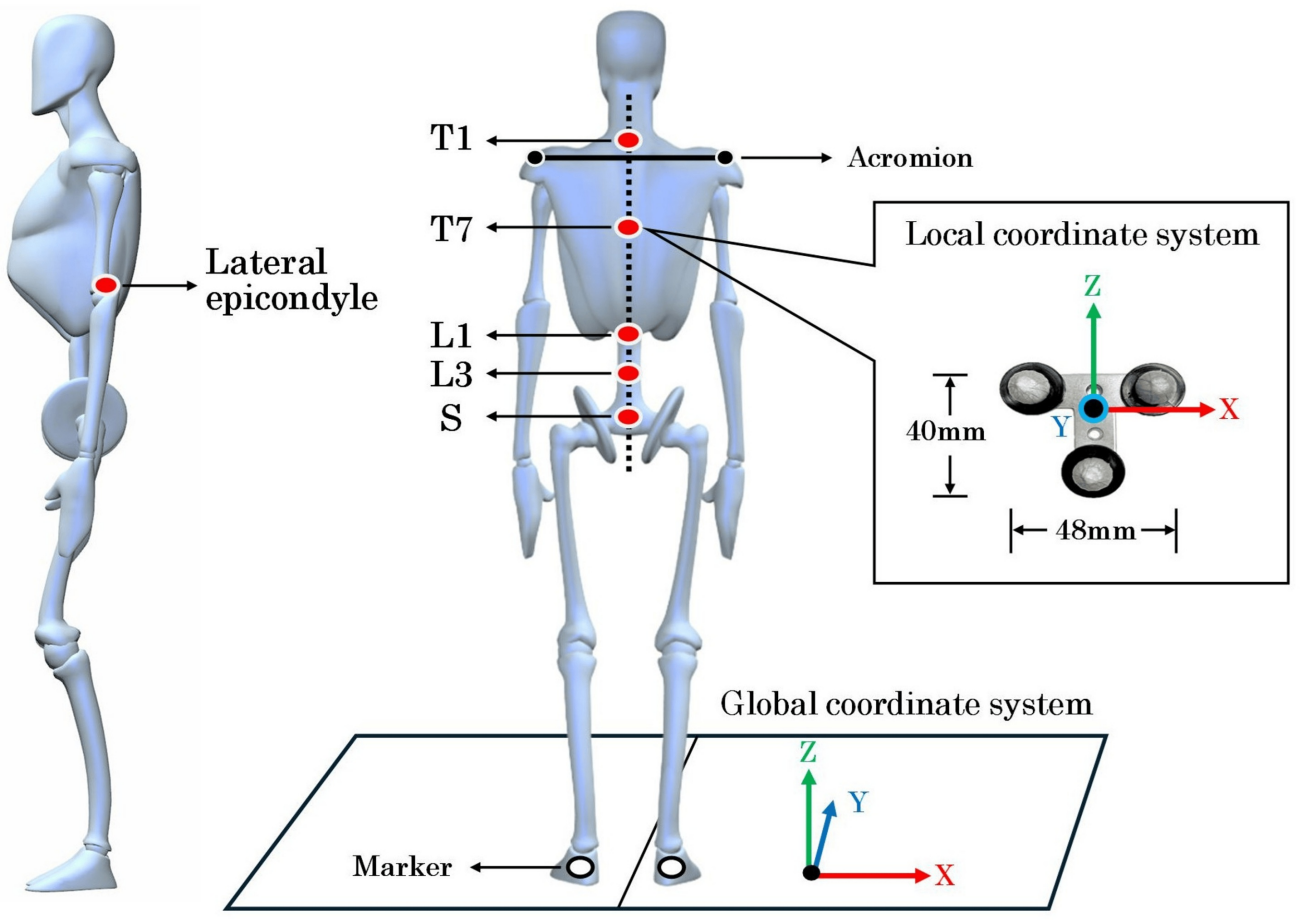
Placement of the T-shaped plates T1: the spinous process one level below the maximum prominence when the neck is flexed. T7: the midpoint of the line connecting the inferior angles of both scapulae. L1: the spinous process two levels above the L3 spinous process. L3: the spinous process one level above the L4 spinous process, identified from the midpoint of Jacoby’s line (L4/L5 interval). S: the midpoint of the line connecting the posterior superior iliac spines. Lateral epicondyle: the most prominent bony protuberance on the lateral aspect of the distal humerus. The image was created using Poser 12. Image Credits: Tsubasa Yamaguchi.

Seven T-shaped stainless steel plates (T-plates, 48 mm in width and 40 mm in height) were used. Markers (φ9 mm) were affixed at three points on the tips of each T-plate, following the method described by Morikawa et al. [16]. The seven T-plates were attached to the first thoracic spinous process (T1), the seventh thoracic spinous process (T7), the first lumbar spinous process (L1), the third lumbar spinous process (L3), the second sacral spinous process (S), and the lateral epicondyles of both upper arms (Figure 3).

The T-plates for the spine were affixed to the measurement bodysuit with double-sided tape. Ensuring that their long axis was as parallel to the floor as possible and closely aligned with the line connecting both acromions, while their short axis was in line with the vertical line from the spinous processes to the floor. The T-plates for the lateral epicondyles were attached so that their short axis aligned with the long axis of the humerus. Additionally, a single marker was attached to each calcaneus to detect the gait cycle.

Participants walked on the treadmill for 110 s, including a 20 s acceleration phase followed by a 90 s steady-state phase. The walking speed during the steady-state phase was set at 4.0 km/h, which is a comfortable walking speed [17]. Participants performed a 5-minute treadmill walking practice session before a single trial of the primary measurement. They were instructed to gaze at a target mark on the wall 5 m ahead while walking.

Data analysis

The parameters analyzed consisted of the relative rotational angles of each T-plate in the horizontal plane and the rotational angles of the left and right lateral epicondyles of the humerus in the sagittal plane. These rotational angles were determined as Euler angles with an XYZ rotation order, where the X-axis corresponds to the lateral direction of the long axis and the Z-axis corresponds to the vertical direction of the short axis. This analysis was conducted using the motion analysis software Body Builder (Body Builder Version 3.6.4, Vicon Motion Systems Ltd., Oxford, UK). The sampling frequency was set at 100 Hz, and data were processed using low-pass filtering (Butterworth filter) at 6 Hz.

Spinal levels were classified as follows: upper thoracic spine (T1-T7), lower thoracic spine (T7-L1), upper lumbar spine (L1-L3), and lower lumbar spine (L3-S). The relative rotational angle of each spinal level was defined as the rotation of the superior vertebra around the Z-axis of the local coordinate system of the inferior vertebra. These angles were denoted as the T1-T7, T7-L1, L1-L3, and L3-S angles. The maximum change in the rotation angle during a gait cycle (rotation angle) was calculated. In line with a previous study [18], arm swing magnitude was defined as the rotational angle of the lateral epicondyles of both upper arms around the X-axis in the global coordinate system. The arm swing angle, which is the maximum change in the rotation angle during a gait cycle, was calculated. The gait cycle was identified by detecting the initial ground contact marked by the minimum Z-axis position of the markers attached to the left and right calcaneus within one complete cycle [17]. Data on the last 10 gait cycles within the final 30 seconds of the steady-state phase were extracted for analysis. These data were time-normalized to one gait cycle and averaged using an additive mean approach.

Statistical analysis

Statistical analysis was performed using IBM Corp. Released 2023. IBM SPSS Statistics for Windows, Version 29.0.2.0 Armonk, NY: IBM Corp. Data normality was assessed using the Shapiro-Wilk test. Differences in arm swing angles between the left and right arms were evaluated using a paired t-test. Spinal rotation angles were compared among different spinal levels using repeated measures ANOVA and multiple comparison tests with Bonferroni correction as a post hoc test. The effect size (r) was calculated for both tests, with r < 0.1 indicating negligible, r = 0.1-0.3 indicating small, r = 0.3-0.5 indicating medium, and r > 0.5 indicating large [19]. The relationship between arm swing angles and rotation angles at each spinal level was investigated using Pearson’s product-moment correlation coefficient. A *p*-value < 0.05 indicated statistical significance. Further, the sample size was calculated using statistics software (G*Power 3.1.9.7, Heinrich Heine University, Düsseldorf, Germany). The significance level was set at α = 0.05, power was set at 1−β = 0.8, and the effect size (r) was assumed to be 0.66, based on the average effect size of spinal rotation angles at each level during steady-state walking obtained in the preliminary study, and the minimum number of participants in the group was recommended to be 21.

## Results

Characteristics of handedness

The FLANDERS handedness questionnaire results indicated an average score of 9.9 (with a standard deviation of) ± 0.3.

Arm swing angle

The left arm swing angle was significantly higher (*p* < 0.05) than the right arm swing angle (Table 1). For 18 of the 21 participants, the swing angle of the left arm was more prominent than that of the right arm.

**Table 1:** Differences in arm swing angles between the left and right arms *p < 0.05. Data are presented as mean ± SD. 95% CI: 95% confidence interval. Effect size: r.

Dominant arm swing side	Right arm swing angle (°)	Left arm swing angle (°)	Mean difference (°)	95% CI lower	95% CI upper	p	Effect size
3 Right/ 18 Left	28.6 ± 11.5	33.1 ± 11.0	-4.5	-8.0	-4.0	0.01*	0.52

Spinal rotation angles at each level

The repeated measures ANOVA revealed a significant main effect (*p* < 0.05) for the spinal rotation angles at each level. Post hoc tests showed that the T1-T7 angle was significantly lower (*p* < 0.05) than the T7-L1, L1-L3, and L3-S angles. Additionally, the L3-S angle was significantly lower (*p *< 0.05) than the T7-L1 and L1-L3 angles (Table 2).

**Table 2:** Spinal rotation angles at each level *p < 0.05. Data are presented as mean ± SD. 95% CI: 95% confidence interval. Effect size: r.

Each spinal level	Angle (°)	p			
T1–T7	3.4 ± 1.4	< 0.001*			
T7–L1	9.1 ± 4.2				
L1–L3	8.9 ± 2.7				
L3–S	4.8 ± 1.8				
Comparison					
Each spinal level	Mean difference (°)	95% CI lower	95% CI upper	p	Effect size
T1–T7 vs T7–L1	-5.7	-7.6	-3.9	< 0.001*	0.82
T1–T7 vs L1–L3	-5.5	-6.7	-4.3	< 0.001*	0.91
T1–T7 vs L3–S	-1.4	-2.3	-0.6	< 0.001*	0.63
T7–L1 vs L1–L3	0.2	-2.4	2.8	0.872	0.04
T7–T1 vs L1–L3	4.3	2.4	6.5	< 0.001*	0.73
L1–L3 vs L3–S	4.1	2.5	5.0	< 0.001*	0.83

Relationship between arm swing angles and spinal rotation angles at each level

**Figure 4:**
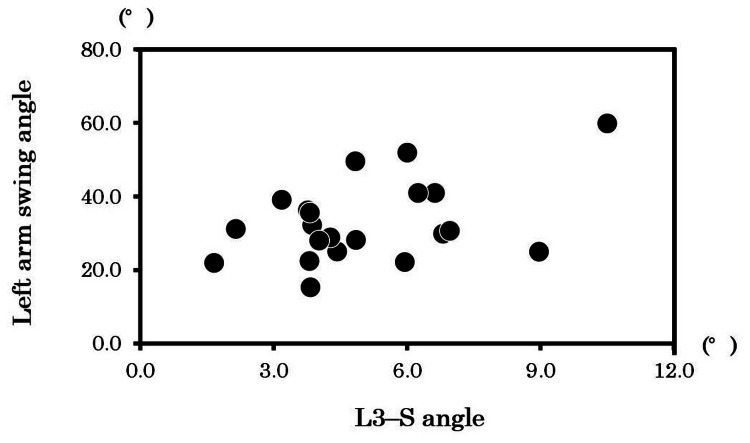
Relationship between the left arm swing angle and the L3–S angle *p < 0.05. *r* = 0.50.

The correlation analysis between the left and right arm swing angles and spinal rotation angles at each level revealed that the left arm swing angle was significantly positively correlated with the L3-S angle (*r* = 0.50, *p* < 0.05), as shown in Table 3 and Figure 4.

**Table 3:** Relationship between arm swing angles and spinal rotation angles at each level *p < 0.05. correlation coefficient: *r* = 95% CI: 95% confidence interval.

Variable	Angle (deg)	r	95% CI lower	95% CI upper	p
Left arm swing angle (°)	T1–T7 angle	0.16	-0.30	0.55	0.50
	T7–L1 angle	-0.31	-0.65	0.15	0.17
	L1–L3 angle	0.34	-0.21	0.67	0.13
	L3–S angle	0.50	0.11	0.76	0.02*
Right arm swing angle (°)	T1–T7 angle	0.37	-0.26	0.57	0.10
	T7–L1 angle	-0.09	-0.50	0.36	0.67
	L1–L3 angle	0.14	-0.31	0.54	0.55
	L3–S angle	0.38	-0.07	0.69	0.08

## Discussion

This study investigated the relationship between arm swing angles and rotation angles at each spinal level during steady-state walking. The results showed that the left arm swing angle was greater than the right arm swing angle. Furthermore, the rotation angle at the T1-T7 level was the lowest among all spinal levels. The analysis of the relationship between arm swing angles and rotation angles at each spinal level revealed a significant positive correlation between the left arm swing angle and the L3-S angle.

Characteristics of handedness

The FLANDERS handedness questionnaire indicated an average score of 9.9 ± 0.3. According to a previous study, scores between 5 and 10 are classified as demonstrating right-handedness [14], which suggests that the participants in this study exhibited a strong tendency toward right-handedness.

Arm swing angle

During steady-state walking, the right arm swing angle was 28.6 ± 11.5°, whereas the left arm swing angle was 33.1 ± 11.0°, indicating that the left arm swing angle was significantly greater than the right arm swing angle. Furthermore, in 18 out of 21 participants, the left arm swing angle was more pronounced than the right arm swing angle. These findings are consistent with previous studies [6-8]. On the other hand, previous studies have shown no significant association between arm swing asymmetry and handedness [18,20]. Other factors contributing to arm swing asymmetry have been identified, such as environmental influences from a social background where right-handedness is more common [20] as well as preference for a dominant foot [21]. These findings imply that the asymmetry in arm swing angle may be affected by several factors beyond handedness.

Spinal rotation angles at each level

The comparison of rotation angles at each spinal level showed significant differences, with the T1-T7 angle being the lowest (T1-T7 angle < L3-S angle < L1-L3 angle < T7-L1 angle). Previous research has indicated that the rotation angle of the thoracic spine (from the seventh cervical vertebra to the first lumbar vertebra) is 8.9° and that of the lumbar spine (from the first lumbar vertebra to the first sacral vertebra) is 11.0 [22]. The rotation angle for the thoracic (T1 to L1) and lumbar (L1 to S) sections obtained in this study were largely consistent with these previous findings, demonstrating high reliability. Additionally, the novelty of this study lies in its approach of dividing the spine into upper, middle, and lower regions and clarifying the order of the magnitude of the range of motion among different spinal levels. These findings indicate that spinal rotation angles exhibit distinct kinematic characteristics at each spinal level and may play different roles in motor control during walking. The T1-T7 and L3-S levels enhance spinal stability and support functions by limiting rotational angles, whereas the T7-L1 and L1-L3 levels provide greater mobility by allowing larger rotational angles. In other words, each region of the spine adjusts its range of motion in a trade-off manner to strike a balance between stability and mobility, specifically, between dynamic stability and mobility [23,24]. Particularly, the T1-T7 rotation angle was significantly lower than that of other spinal levels, which may be an important factor in maintaining overall body stability during walking [25]. The restriction of rotational movement at the T1-T7 level may contribute to head stability and maintenance of forward gaze by mitigating vibrations during walking. Additionally, the stabilization of the T1-T7 and L3-S regions may facilitate the increased rotational motion seen at the T7-L1 and L1-L3 levels. This functional distribution of the spinal rotation angles at various levels is believed to contribute to the efficient transfer of energy between the movement of the upper and lower limbs during walking [26].

Relationship between arm swing angles and spinal rotation angles at each level

This study showed a significant positive correlation (*r* = 0.50) between the left arm swing angle and the L3-S angle. This result can be interpreted in light of the passive model, which proposes that the energy generated by the alternating stepping motion of the lower limbs during walking is transmitted from the pelvis through the spine to the glenohumeral joint, thereby inducing arm swing [10,27]. According to the passive model, the L3-S angle may be a crucial intermediary point for energy transfer leading to arm swing.

However, no significant correlation was observed between the arm swing angles and the T1-T7, T7-L1, or L1-L3 angles. These findings indicate that arm swing cannot be fully explained by energy transmission through the entire spine. Regarding the mechanism of arm swing generation, the active model proposes that the activity of the muscles around the upper limbs drives arm swing [28-30]. This indicates that arm swing is influenced by spinal rotation and other contributing factors. Recent studies have shown that arm swing is modulated by the interaction between spinal rotation and muscle activity in the upper limbs [27,28,31]. Therefore, arm swing is not entirely dependent on spinal rotation but may be influenced by multiple other factors.

Limitations

This study has some limitations. First, the measurement task involved walking on a treadmill, which may have led to movement strategies different from those used in overground walking [32]. Second, the study included young, healthy males, which may limit the generalizability of the findings. Therefore, further studies involving female participants and individuals from a broader age range are needed.

## Conclusions

This study showed that the arm swing angle of the left arm was significantly greater than that of the right arm. Furthermore, the rotation angle at the T1-T7 level was the lowest, indicating that each level exhibits distinct kinematic characteristics. Additionally, a significant positive correlation was observed between the left arm swing angle and the L3-S angle. These findings indicate that the left arm swing during steady-state walking is locally influenced by the rotational movement at the L3-S level. However, the arm swing is not entirely dependent on spinal rotation, and multiple other factors likely affect it.
